# Serum Spexin Level Is Negatively Associated With Peripheral Neuropathy and Sensory Pain in Type 2 Diabetes

**DOI:** 10.1155/2024/4538199

**Published:** 2024-05-23

**Authors:** Ying Liu, Di Wu, Hangping Zheng, Yunzhi Ni, Lu Zhu, Yaojing Jiang, Jiarong Dai, Quanya Sun, Ying Zhao, Qi Zhang, Yehong Yang, Rui Liu

**Affiliations:** ^1^ Department of Endocrinology and Metabolism Huashan Hospital Fudan University, Shanghai 200040, China; ^2^ Institute of Endocrinology and Diabetes Fudan University, Shanghai 200040, China

**Keywords:** diabetic peripheral neuropathy, pain, PBMCs, spexin

## Abstract

**Background:** Spexin is a novel peptide hormone and has shown antinociceptive effects in experimental mice. This study is aimed at evaluating the association of serum spexin level with diabetic peripheral neuropathy (DPN) and related pain in a Chinese population.

**Methods:** We enrolled 167 type 2 diabetes mellitus (T2DM) including 56 patients without DPN (non-DPN), 67 painless DPN, and 44 painful DPN. Serum spexin was measured using ELISA. Logistic regression models were performed to analyze the independent effects of spexin on prevalence of DPN and painful DPN. In streptozotocin (STZ)-induced diabetic mice, mechanical pain threshold was measured using electronic von Frey aesthesiometer. Human peripheral blood mononuclear cells (PBMCs) were isolated and further stimulated with lipopolysaccharide without or with spexin. The gene expression was assayed by qPCR.

**Results:** Compared with non-DPN, serum spexin level decreased in painless DPN and further decreased in painful DPN. The odds of DPN was associated with low spexin level in T2DM, which was similar by age, sex, BMI, and diabetes duration, but attenuated in smokers. The odds of having pain was associated with decreased spexin level in DPN, which was similar by age, sex, smoking status, and diabetes duration, but attenuated in normal weight. Furthermore, we observed that mechanical pain threshold increased in spexin-treated diabetic mice. We also found that lipopolysaccharide treatment increased the mRNA level of TNF-*α*, IL-6, and MCP-1 in human PBMCs, while spexin treatment prevented this increase.

**Conclusions:** These results suggested that spexin might serve as a protective factor for diabetes against neuropathology and pain-related pathogenesis.

## 1. Introduction

Diabetic peripheral neuropathy (DPN) is the most common and intractable complication of diabetes [1]. DPN patients experience sensory symptoms in the feet, such as pain, tingling, prickling sensations, and numbness [2]. Among these symptoms, pain is often severe and challenging to manage due to its relationship to sleep disturbances and depression [3]. In addition, DPN is the key initiating factor for the development of diabetic foot ulceration, which is the most common cause of nontraumatic lower-limb amputations [4]. However, there is no clear pathophysiological explanation for DPN and related symptoms. The sensory nerves emerge from the dorsal root ganglion (DRG), which conveys the sensations from the peripheral nervous system to the central nervous system [5]. Both the peripheral and central nervous systems are supposed to be involved in the DPN progression [6].

Spexin, also referred to as neuropeptide Q, is a recently discovered neuropeptide [7, 8]. The sequence of the mature peptide of spexin is constituted of 14 amino acids, which is evolutionally conserved in vertebrates [9, 10]. Spexin is present in various brain regions and peripheral tissues [11, 12], and its functional role has been associated with feeding behavior, obesity, mood, and nociception [9, 13]. Spexin treatment shows improvement on both inflammatory pain and visceral pain in mouse models [14, 15].

Spexin is phylogenetically related to galanin and classified as a member of galanin family [10]. Galanin plays an important role in neural generation and nociception. Galanin acts as a trophic factor to the central and peripheral nervous systems [16] via activation of galanin receptor 2 [17]. The effects of galanin on nociception are receptor subtype-specific. A low dose of galanin has a nociceptive role at the spinal cord level mediated by galanin receptor 2, whereas the antiallodynic effect of high-dose galanin on neuropathic pain is mediated by galanin receptor 1 [18]. Studies have shown that spexin preferentially binds to galanin receptors 2 and 3, but not galanin receptor 1 [19]. Furthermore, the antinociception of spexin against inflammatory pain can be blocked by the antagonist of galanin receptor 3 [15].

Despite the compelling data in animal studies, to our knowledge, the association of serum spexin with DPN and sensory pain in diabetes has not been investigated. Clinical studies also document that type 1 diabetes mellitus (T1DM) and type 2 diabetes mellitus (T2DM) have lower levels of serum spexin [20, 21]. Thus, we hypothesize that insufficiency of spexin in diabetes might increase susceptibility to DPN and pain. In the current study, we first examined the association between serum spexin level and the odds of prevalence of DPN in diabetes and pain in DPN. Based on this clinical relationship, we then manipulated the spexin level in a diabetic mouse model to test the effect of spexin on pain symptoms and explored the potential mechanism.

## 2. Methods

### 2.1. Study Subjects

A total of 167 patients with T2DM were recruited in this study, including 56 diabetes without DPN (non-DPN), 67 painless DPN, and 44 painful DPN. The sample size calculation was performed based on a study reporting the difference in spexin level in cataract patients with diabetes in the presence or absence of retinopathy [22]. T2DM diabetes was defined according to WHO 1999 criteria. The assessment of DPN was performed fulfilling the China Diabetic Neuropathy Expert Consensus based on Toronto criteria for a confirmed diagnosis of DPN [23].

Patients underwent nerve conduction studies (NCSs) and were evaluated for symptoms and signs predominantly in the toes, feet, legs, or arms that could indicate DPN. NCS was conducted using an electromyography machine (Keypoint 9033A07, Focus Company, Denmark) on sural, common peroneal, tibial, median, and ulnar nerves of the affected side. Symptoms of DPN included decreased sensation and positive neuropathic sensory symptoms such as pain, numbness, and sensory abnormalities. Clinical signs of DPN were evaluated in the feet or arms by five tests: (1) activity of ankle reflexes with reinforcement applied. If the reflex of two feet decreased or did not appear, it was considered abnormal; (2) vibration sensation at the dorsal aspect of the great toes using a 128-Hz tuning fork and the on–off method. If the sensation of either foot disappeared, it was considered abnormal; (3) light touch sensation by a 10-g monofilament on the dorsal aspect of the great toes. The test was repeated for four times on each side, and the total number of times no pressure was recorded for scoring as 1 point per time. If ≥ 5 points, it was considered abnormal; (4) acupuncture pain sensation by a large needle on the patient's foot skin. If the sensation of either foot disappeared, it was considered abnormal; (5) temperature sensation by examination instruments with the metal end that felt cool and the polymer that felt hot. If the patient could not distinguish the temperature difference between the two ends in either foot, it was considered abnormal. Patients with abnormalities in NCS and at least one symptom or sign of DPN were diagnosed with confirmed DPN.

After the diagnosis of DPN, patients were further classified into painless and painful groups based on their descriptions of pain symptoms. Patients with painful DPN typically reported distal, symmetrical pain, often associated with nocturnal exacerbations. The pain was commonly described as prickling, deep aching, sharp, electric shock like, or burning. To ensure the specificity of the painful DPN diagnosis, other potential causes of neuropathic pain and leg/foot pain, such as peripheral vascular disease, arthritis, malignancy, alcohol abuse, and spinal canal stenosis, were carefully excluded from the study.

The following exclusion criteria were used in this study: peripheral neuropathy of nondiabetic origin; acute or chronic inflammatory disease; heart, liver, or renal failure; cancer; or pregnancy.

All patients had been receiving antihyperglycemic therapy with more than one class of medicine. The antihyperglycemic medicines are mainly insulin and oral antihyperglycemic agents including sulfonylureas, biguanides, and dipeptidyl peptidase IV (DPP-IV) inhibitors. A few painful DPN patients received analgesic medicines such as pregabalin and gabapentin. All patients with T2DM had at least one comorbidity, such as thyroid nodules, hypertension, and pulmonary nodules.

The study was approved by the human research ethics committee of the Huashan Hospital in Shanghai, which conforms to the provision of the Declaration of Helsinki (as revised in Fortaleza, Brazil, October 2013). All participants provided written informed consent before participating in this study.

### 2.2. Clinical Measurements

All patients completed a questionnaire and underwent a detailed anthropometric assessment to collect demographic and clinical data, including age, sex, diabetes duration, and smoking history. Participants' weight (in light clothing) and height (without shoes) were measured, and the body mass index (BMI) was calculated as weight in kilograms divided by the square of the height in meters. Laboratory investigations were done with blood samples collected from the antecubital vein following 8–12 h of overnight fasting. Blood cell count, liver function, kidney function, fasting blood glucose, serum creatinine (sCr), hemoglobin A1c (HbA1c), and fasting insulin (FINS) were analyzed. Glomerular filtration rate was estimated from sCr using the re-expressed Modification of Diet in Renal Disease equation defined as follows: Estimated glomerular filtration rate (eGFR) = 175 × (sCr in mg/dl)^−1.154^ × age^−0.203^ × (0.742 for women). The homeostatic model assessment for insulin resistance (HOMA2-IR) and homeostatic model assessment for *β* cell function (HOMA2-*β*) was calculated from fasting plasma glucose (FPG) and C-peptide concentrations by using the updated HOMA2 calculator (version 2.2.4; https://www.dtu.ox.ac.uk/).

### 2.3. Serum Spexin Measurement

Blood samples were collected from the patients. Serum was separated after centrifugation at 3000 g for 10 min and stored at −80°C. Serum spexin level was measured using an ELISA kit (Catalog # EH4349, Fine Biotech Co., Wuhan, Hubei, China) as previously described [21]. The intra- and interassay CV is < 8% and < 10%, respectively. This assay has high sensitivity and excellent specificity for detection of spexin. No significant cross-reactivity or interference between spexin and galanin, kisspeptin, insulin, and C-peptide (all 25 ng/ml) was observed.

### 2.4. Mice and Behavior Test

Eight-week-old C57BL/6 male mice were intraperitoneally (i.p.) injected without or with streptozotocin (STZ) at 50 mg/kg body weight (BW) for 5 days. Blood glucose level ≥ 16.7 mmol/L was considered as diabetic. The mice without STZ injection were normoglycemia (NG). Eighteen weeks after STZ injection, the diabetic mice were further divided into two groups: (1) diabetes-saline (DM/Saline) and (2) diabetes-spexin (DM/Spexin). The mice in the DM/Spexin group were i.p. injected with 200 *μ*g/kg BW spexin. Mechanical pain threshold was measured before and after spexin injection.

The mechanical nociceptive threshold was assessed by an electronic von Frey apparatus as previously described [24]. Briefly, mice were placed in acrylic cages with a wire grid flooring and allowed to acclimate for 30 min. The single filament was applied to slowly stimulate the left hind paw, and the force gradually increased. When mice responded with hind paw retraction or licking, the force was recorded as the threshold. Each trial was repeated three times with 15-min intervals between measurements. For each mouse, the results of three measurements were averaged for subsequent statistical analysis. The paw withdrawal threshold assessment was conducted by two investigators to ensure blinding. On the day of testing, one investigator was responsible for the injection and recording data. The other investigator applied the von Frey filament to the hind paw of mice and observed the response of the mice. All experimental mice were approved according to the Institutional Animal Care and Use Committee of Huashan Hospital, Fudan University (Shanghai, China).

### 2.5. Immunofluorescence

The immunofluorescence was performed as previously described [25]. DRG from normal mice was fixed overnight in 4% paraformaldehyde and then embedded in paraffin. The paraffin blocks were cut into sections with 6–7 *μ*m thickness. The tissue sections were immunostained with primary antibodies against galanin receptor 2 (1:100, 26459; Proteintech, China) and neuronal nuclear protein (NeuN) (1:200, 66836; Proteintech, China) overnight at 4°C followed by incubation with secondary antibodies for 1 h at room temperature. All images were captured using a Lexia microscope.

### 2.6. Isolation and Culture of Human Peripheral Blood Mononuclear Cells (PBMCs)

Human PBMCs were isolated using human PBMC isolation kit (TBD2011H; Haoyang Biological Manufacture Co., Ltd., China) following the manufacturer's instructions. Separation solution 1, separation solution 2, and blood were loaded in sequence. After centrifugation at 600 g for 30 min, the cells at second gradient from the top were collected and recovered in RPMI-1640 supplemented with 10% FBS for 6 h before treatment.

### 2.7. RNA Extraction and Real-Time Quantitative Polymerase (qPCR)

Total RNA was extracted from PBMCs using TRIzol reagent (Invitrogen; Thermo Fisher Scientific Inc., USA) according to the manufacturer's instructions. The RNA purity and concentration was measured using a NanoDrop system (NanoDrop Technologies, USA). Total RNA (1 *μ*g) was reverse transcribed to cDNA using the Hifair® III 1st Strand cDNA Synthesis SuperMix for qPCR (11141ES10; Yeasen, China). The cDNA production was diluted five times and then used as the template for PCR using Hieff UNICON qPCR SYBR Green Master Mix (11198ES08; Yeasen, China). The relative expression level of genes was calculated by the comparative 2^−*ΔΔ*CT^ method with the housekeeping gene GAPDH for normalization. The sequences of primers were as follows: tumor necrosis factor-*α* (TNF-*α*) (forward, ACCTGGCCTCTCTACCTTGT; reverse, CCCGTAGGGCGATTACAGTC), interleukin-6 (IL-6) (forward, CAACGATGATGCACTTGCAGA; reverse, TCTCTCTGAAGGACTCTGGCT), and monocyte chemoattractant protein-1 (MCP-1) (forward, ACCAGCCAACTCTCACTGAA; reverse, GCCAGTGAATGAGTAGCAGC).

### 2.8. Statistical Analysis

Clinical data were tested for normality using the Kolmogorov–Smirnov test before statistical analysis. Normally distributed continuous variables were expressed as mean ± standard deviation (SD). Skewed distributed continuous variables were logarithmically transformed and expressed as geometrical median and interquartile range (median [P25, P75]). Categorical variables were expressed as frequencies and proportions. Continuous variables were compared by the Student *t* test and one-way analysis of variance (ANOVA). Categorical variables were compared using chi-square tests. All analyses for multiple comparisons were corrected using Bonferroni correction.

Spearman's correlation analysis was performed for spexin and the remaining variables. Spexin level was treated as a categorical variable (by tertiles). Linear regression analyses were applied for the association of spexin and the clinical characteristics of patients. Logistic regressions were used to estimate odds ratios (ORs) and 95% confidence intervals (CIs) of the presence of DPN and painful DPN, with the lowest tertile as the reference. The subgroup analyses of gender (male vs. female), age (< 65 vs. ≥ 65 years), BMI (< 24 vs. ≥ 24 kg/m^2^), diabetes duration (< 10 vs. ≥ 10), and tobacco use (nonuser vs. user) were further explored to test if the results were consistent among different subgroups. *P* for heterogeneity was analyzed by Comprehensive Meta-Analysis (CMA) software version 3.0 (Biostat Inc., Englewood, NJ, USA). All statistical tests used the two-tailed method.

For experiments on animal and human PBMCs, data are presented as mean ± SD. Statistical analysis was carried out using unpaired Student's *t* test. GraphPad Prism 9.0 software (GraphPad, San Diego, CA, USA) was used for statistical analysis. *P* value < 0.05 was considered statistically significant.

## 3. Results

### 3.1. Demographics and Diabetic Characteristics

Table 1 summarizes the demographic and diabetic characteristics performed for each group. There were no differences in sex, smoking status, eGFR, neutrophil-to-lymphocyte ratio (NLR), alanine aminotransferase (ALT), FPG, HbA1c, FINS, fasting C-peptide, HOMA2-IR, and HOMA2-*β*. The patients in non-DPN were the youngest (*P* < 0.05), experienced the shortest duration of diabetes (*P* < 0.01), and had the lowest prevalence of diabetic retinopathy (*P* < 0.001). Painful DPN patients had lowest levels of BMI and aspartate aminotransferase (AST) (both *P* < 0.05). All patients had at least one comorbidity, and there was no difference in the prevalence of comorbid hypertension, cerebrovascular accident (CVA) or coronary heart disease (CHD) among non-DPN, painless DPN, and painful DPN.

### 3.2. Serum Spexin Level

**Figure 1:**
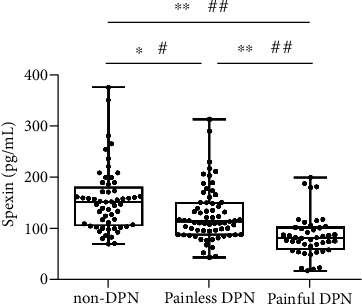
Serum spexin concentrations (median [min to max]) in patients with non-DPN (*n* = 55), painless DPN (*n* = 67), and painful DPN (*n* = 44). Spexin level is lower in painless DPNs compared to non-DPNs and further decreases in painful DPNs. Spexin is log transformed for statistical analysis. ^∗^*P* < 0.01, ^∗∗^*P* < 0.001, unadjusted; ^#^*P* < 0.05, ^##^*P* < 0.001, after age, gender, and BMI adjustment.

In a total of 167 subjects, serum level of spexin ranged from 17.21 to 350.00 pg/mL (Figure 1). Spearman's correlation analyses revealed that spexin level had a positive correlation with levels of AST (*r* = 0.2, *P* < 0.01), C-peptide (*r* = 0.277, *P* < 0.001), HOMA2-IR (*r* = 194, *P* < 0.05), and HOMA2-*β* (*r* = 0.272, *P* < 0.001) (Table S1). Compared with non-DPN (149.16 pg/mL [105.51, 172.45]), serum spexin level decreased in painless DPN (113.47 pg/mL [87.99, 149.92]) (*P* < 0.01) and further decreased in painful DPN (81.45 pg/mL [59.83, 101.46]) (*P* < 0.001). After adjustment for age, sex, and BMI, the difference was still significant in painless DPN compared to non-DPN (*P* < 0.05) and painful DPN compared to other groups (both *P* < 0.001).

### 3.3. Correlation Between Spexin Level and Odds of Prevalent DPN in All Diabetes

All participants were categorized according to tertiles of fasting serum spexin concentrations (Table S2 and Table 2). Fasting plasma AST, ALT, C-peptide, HOMA2-IR, and HOMA2-*β* increased following increasing serum spexin tertiles (all *P* < 0.05), whereas age decreased following increasing serum spexin tertiles (Table S2, *P* < 0.05). A graded decrease in prevalent DPN was observed with an increase in fasting serum spexin tertiles (Table 2, *P* < 0.001). Furthermore, logistic regression models were performed to investigate the effect of fasting serum spexin level on prevalent DPN in a series of adjusted models (Table 2). In the age, sex, and BMI adjusted model, compared with participants in the lowest tertile, participants in the second tertile had a 71% (OR 0.29 [95% CI 0.11–0.80]) lower odds of prevalence of DPN, while participants in highest tertile had an 89% (OR 0.11 [95% CI 0.04–0.28]) lower odds of prevalence of DPN (*P* < 0.001). The association was slightly attenuated but still remained after additional adjustment for duration of DM, C-peptide, and smoking status (*P* < 0.001).

**Figure 2:**
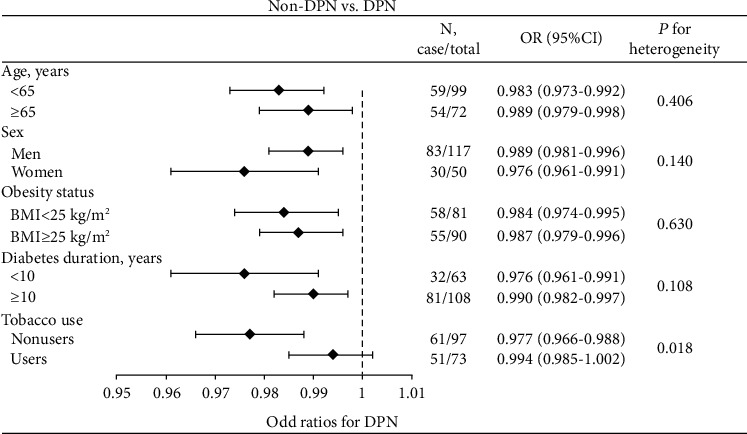
Subgroup analyses of OR for the association between fasting serum spexin level and risk of having DPN.

Next, we addressed the differences in the association between fasting serum spexin level and the prevalence of DPN among the subgroups according to age, sex, obesity status, diabetes duration, and smoking status (Figure 2). The association was similar in individuals aged < 65 or ≥ 65 years, men or women, with BMI < 25 kg/m^2^ or ≥ 25 kg/m^2^, and diabetes duration < 10 or ≥ 10 years (all *P* > 0.10). However, the association was stronger in nonsmokers than smokers (Figure 2, *P* = 0.018).

### 3.4. Correlation Between Spexin Level and Odds of the Presence of Painful Symptoms in DPN

The DPN patients were categorized according to tertiles of fasting serum spexin level (Table S3 and Table 3). AST and HOMA2-*β* increased following increasing serum spexin tertiles (Table S3, all *P* < 0.05). A decrease in the prevalence of painful DPN was observed with increasing serum spexin level (Table 3). Compared with the lowest tertile of spexin (referent), the OR (95% CI) of painful DPN was 0.20 (0.08–0.54) in the second tertile and 0.08 (0.03–0.25) in the highest tertile (*P* < 0.001). Further logistic regression analysis showed no attenuation of the association between the spexin level and prevalent painful DPN with additional adjustment for age, sex, BMI, duration of DM, C-peptide, and smoking status (all *P* < 0.001).

**Figure 3:**
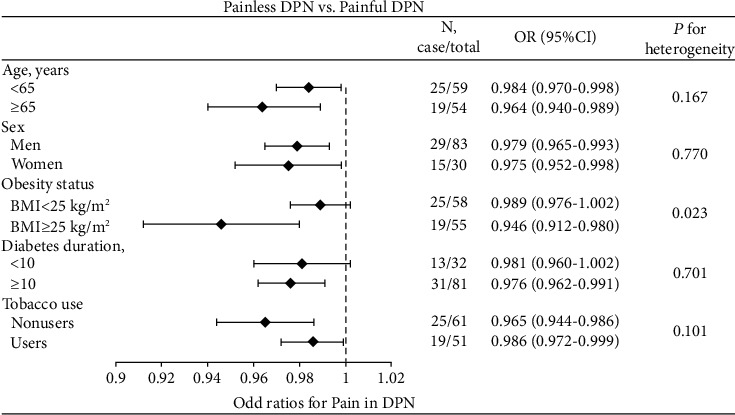
Subgroup analyses of OR for the association between fasting serum spexin level and risk of having pain in DPN patients.

Subgroup analysis demonstrated that the association was similar in individuals aged < 65 or ≥ 65 years, men or women, smokers or nonsmokers, and diabetes duration < 10 or ≥ 10 years (all *P* > 0.10) (Figure 3). However, the association was stronger in BMI ≥ 25 kg/m^2^ than BMI < 25 kg/m^2^ (*P* = 0.023).

### 3.5. Spexin Treatment Increases the Mechanical Nociceptive Threshold of Diabetic Mice

**Figure 4:**
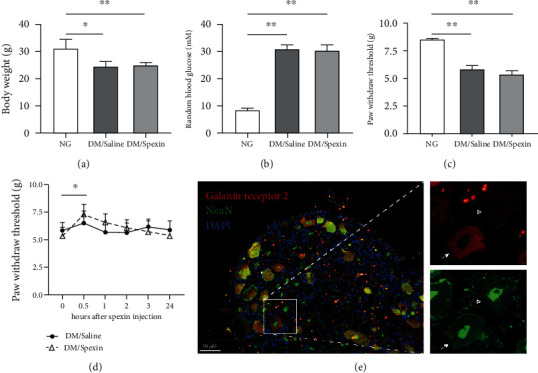
Spexin increases mechanical nociceptive threshold of diabetic mice. (a) BW, (b) random blood glucose, and (c) mechanical pain thresholds of mice at eighteen weeks after STZ injection. (a–c) ^∗^*P* < 0.05 and ^∗∗^*P* < 0.01. (d) Mechanical pain threshold of diabetic mice before and after spexin (200 *μ*g/kg BW) injection. ^∗^*P* < 0.05, DM/Spexin at 30 min vs. DM/Spexin at 0 min. (e) Representative images of immunofluorescence staining of galanin receptor 2 (red) and NeuN (green). The nuclei were stained with DAPI (blue). An arrow indicates galanin receptor 2^+^/NeuN^+^ cell. A white right-pointing small triangle indicates galanin receptor 2^−^/NeuN^+^ cell. Scale bar, 50 *μ*m. (a–d) Data are mean ± SD; *n* = 4 per group; unpaired Student's *t* test. Abbreviation: NeuN, neuronal nuclear protein.

To test whether the decreased levels of circulating spexin contribute to mechanical hyperalgesia in patients with diabetes, we treated diabetic mice with spexin and measured the mechanical nociceptive threshold using an electronic von Frey aesthesiometer. Eighteen weeks after STZ injection, diabetic mice exhibited a significant decrease in BW (Figure 4(a), DM/Saline vs. NG, *P* < 0.05; DM/Spexin vs. NG, *P* < 0.01) and a marked increase in random blood glucose level markedly (Figure 4(b), all *P* < 0.01). The diabetic mice also showed decreased paw withdrawal threshold during nociception stimuli (Figure 4(c), *P* < 0.01), indicating the presence of mechanical hyperalgesia in these mice. We then treated diabetic mice with spexin (200 *μ*g/kg BW) by a single i.p. injection. The paw withdrawal threshold was measured before and after injection at 0, 0.5, 1, 2, 3, and 24 h. We observed a quick increase in paw withdrawal threshold at 30 min after spexin injection (Figure 4(d), *P* < 0.05). Together, these results suggest that spexin treatment improves the mechanical hyperalgesia of diabetic mice.

Studies have shown that spexin has the ability to bind galanin receptors 2 and 3. The effects of spexin on anxiety, hepatic steatosis, and bowel movement may be blocked by galanin receptor antagonists [26–28]. We then tested the expression of galanin receptor 2 on mouse DRG neurons. Immunofluorescence staining showed that galanin receptor 2 was colocalized with mature neuronal protein NeuN (Figure 4(e)), indicating that spexin has the potential to directly affect functions of DRG neurons via galanin receptor 2.

### 3.6. Spexin Treatment Decreases Inflammatory Cytokines in Human PBMCs

**Figure 5:**
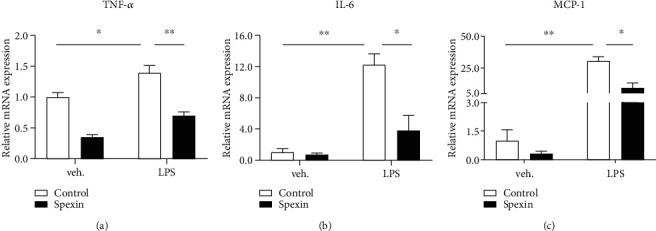
Spexin inhibits the production of inflammatory cytokines in human PBMCs. PBMCs were isolated from three human subjects and then stimulated with LPS (1 *μ*g/mL) without and with 50 nM spexin for 24 h. The mRNA expression of (a) TNF-*α*, (b) IL-6, and (c) MCP-1 was analyzed by qPCR analysis. (a–c) Data are mean ± SD; *n* = 3 per group; unpsaired Student's *t* test. ^∗^*P* < 0.05 and ^∗∗^*P* < 0.01. Abbreviations: LPS, lipopolysaccharide; TNF-*α*, tumor necrosis factor-*α*; IL-6, interleukin-6; MCP-1, monocyte chemoattractant protein-1.

Inflammatory cytokines play an essential role in the development of DPN [29]. To test whether spexin treatment regulates the production of inflammatory cytokines, we stimulated human PBMCs with lipopolysaccharide (LPS) in the presence or absence of spexin for 24 h. LPS (1 *μ*g/mL) treatment significantly increased mRNA level of TNF-*α* (Figure 5(a), *P* < 0.05), IL-6 (Figure 5(b), *P* < 0.01), and MCP-1 (Figure 5(c), *P* < 0.01), while 50 nM spexin treatment prevented this increment (Figures 5(a)–5(c); TNF-*α*, *P* < 0.01; both IL-6 and MCP-1, *P* < 0.05).

## 4. Discussion

In the current study, we discovered a close association between fasting serum spexin level and diabetic neuropathy. In diabetes, the decreased serum spexin level was significantly associated with increased odds of prevalent DPN. Furthermore, in DPN patients, the decreased serum spexin level was associated with increased odds of sensory pain. These associations were independent of established odds factors and appeared to be dose-responsive. To our knowledge, this study evaluated for the first time the association between serum spexin level and the odds of prevalence of DPN in diabetes and sensory pain in DPN.

Several animal studies have shown that spexin plays a protective role in central pain syndrome. Intracranial administration of spexin shows analgesic activity in multiple tests, including the tail-flick test, formalin test, and writhing test, suggesting the antinociceptive effects of spexin against both inflammatory pain and visceral pain [14, 15, 30]. The dependence of these effects on opioid receptors is controversial. The antinociceptive effects of spexin were completely reversed by opioid receptor antagonist naloxone in the formalin test [15], while not in the tail-flick test [30].

We are inspired by our clinical observation that serum spexin was a protective factor for painful DPN. And we further demonstrated that peripheral administration of spexin improved the mechanical hyperalgesia of diabetic mice. As to the paw withdrawal threshold assessed at time points immediately following spexin injection, the antinociceptive effects of spexin we observed are more likely to be direct and potentially mediated through the activation of nociceptors. In addition, we recognize the difference in neuropathy between T1DM and T2DM [31] and the emerging role of metabolic syndrome in the diabetic neuropathy [32]. The STZ-induced diabetic mouse model could not recapitulate the metabolic contributions to neuropathy in T2DM. Therefore, our future research will focus on investigating the effects of long-time spexin treatment on HFD plus STZ-induced diabetic mouse model, which more closely mimics the metabolic alterations associated with T2DM. Furthermore, we observed that some of the DRG neurons expressed galanin receptor 2, which was reported to mediate spexin action in the intestine and colon [28]. Further studies into the contribution of galanin receptor 2 to the diabetic neuropathy and the effects of spexin on pain sensory are required.

Furthermore, we explored the effects of spexin on other pathways in the development and progression of DPN and pain. Chronic low-grade inflammation has been highlighted as one of such critical pathways [33, 34]. The inflammatory mediators may have variations from one person to another and may have variations in the same person at different times. In animal studies, treatments which specifically target macrophages and chemokines are able to reduce neuropathic pain in diabetic mouse models [35, 36]. Spexin treatment has shown to inhibit inflammation by reducing blood IL-6 level in a metabolic syndrome rat model induced by high-fructose diet and blocking macrophage recruitment to adipose tissue in an obese mouse model [37, 38]. In this study, we isolated and stimulated human PBMCs with LPS to produce inflammatory factors. We observed that spexin treatment significantly inhibited LPS-induced expression of IL-6, IL-1*β*, and TNF-*α*. Thus, it is plausible that DPN patients with lower spexin levels have a diminished ability to counter inflammation, resulting in elevated levels of inflammatory factors.

High BMI has been proven to be a risk factor for DPN [39–41]. However, the association between BMI and painful DPN has not been defined. In a small cross-sectional study, high BMI is suggested to be a major determinant of painful DPN [34]. A harmonised cohort (*n* = 1230) built on three large and deeply phenotyped cross-sectional cohorts shows that painful DPN is associated with high BMI [42]. This association still exists in a study with a total of 3021 individuals from the Middle East [43]. However, a study from China enrolling 25,710 DPN patients reports that patients with a BMI > 24 kg/m^2^ have a lower risk and patients with BMI < 18.5 kg/m^2^ have a higher risk than those with normal weight [44]. Thus, this study adjusted BMI in all logistic regression analyses and fully considered the potential impact of BMI.

The association of obesity with incident DPN is partly mediated by inflammatory markers [33]. In our subgroup analysis, the association between spexin level and the presence of painful symptoms in DPN was stronger in BMI ≥ 25 kg/m^2^, suggesting that some metabolic features like inflammation contribute to this relationship.

We have shown that there is a close positive relationship between spexin level and HOMA2-*β* and Stumvoll first-phase insulin secretion and proposed that pancreatic *β* cells are one of the main origins of circulating spexin as previously discussed [21]. This study has shown that there is a positive relationship between C-peptide and serum spexin level. Thus, for long-course diabetic patients, the persistent and irreversible *β* cell loss might account for the decrease in serum spexin level and onset of DPN and pain experience.

The data on the association between spexin level and HOMA-IR is somewhat complicated and mixed. A cross-sectional study in healthy middle-aged women and those with obesity showed a negative correlation between spexin level and HOMA-IR [45]. However, studies in children have showed mixed results with some reporting a negative correlation [46] and others finding no significant correlation [47, 48].

This study is limited by the lack of more comprehensive neurophysiological examinations in the diagnosis of DPN, such as quantitative sensory tests and skin biopsy, both of which can be used to diagnose and evaluate small fiber neuropathy. Other limitations are the lack of appropriate questionnaire for neuropathic pain, the relatively small cohort size, and the cross-sectional design. In summary, we accurately stratified the presence of DPN and painful DPN through detailed clinical and neurophysiological assessments of all participants. Further long-term prospective cohort studies or intervention studies are needed to clarify the specific relationship. It is still unknown whether spexin could pass through the blood-brain barrier and whether spexin had peripherally antinociceptive effects, and further research is needed to explore these mechanisms.

## 5. Conclusions

Our study revealed, for the first time, that serum spexin level decreased in DPN patients and lower serum spexin level was independently associated with the presence of DPN and painful DPN, suggesting the possible protective role of spexin in the neuropathology and pain-related pathogenesis in diabetes.

## Figures and Tables

**Table 1:** Anthropometric parameters and biochemical indexes among subjects with non-DPN, painless DPN, and painful DPN.

**Variables**	**Non-DPN (** **n** = 56**)**	**Painless DPN (** **n** = 67**)**	**Painful DPN (** **n** = 44**)**	**P** **value**
Male, *n* (%)^#^	33 (58.9%)	52 (77.6%)	29 (65.9%)	0.079
Age (years)	57.96 ± 10.52	63.19 ± 10.58^b^	62.41 ± 12.92	0.028
BMI (kg/m^2^)	25.68 ± 3.14	25.28 ± 3.11	24.14 ± 3.50	0.054
Duration (years)^a^	9.00 (2.00, 14.50)	16.00 (9.00, 20.00)^b^	13.00 (8.25, 18.00)^c^	0.004
Smoking, *n* (%)^#^	21 (37.5%)	30 (44.8%)	19 (43.2%)	0.667
NLR^a^	1.84 (1.25, 2.40)	2.48 (1.83, 3.39)	2.20 (1.59, 2.81)	0.172
eGFR (mL/min/1.73 m^2^)^a^	99.07 (93.60, 109.35)	95.86 (77.51, 103.18)	97.34 (84.88, 110.20)	0.134
Diabetic retinopathy, *n* (%)	6 (10.7%)	34 (50.7%)^b^	20 (45.5%)^c^	< 0.001
Liver function				
ALT (U/L)^a^	17.50 (14.00, 32.00)	18.00 (13.00, 24.00)	17.00 (13.00, 24.00)	0.062
AST (U/L)^a^	18.00 (15.00, 22.00)	17.00 (14.00, 21.00)	15.00 (12.00, 20.75)^c^	0.027
Glucose metabolism				
HbA1c (%)	8.96 ± 2.07	9.32 ± 2.25	8.61 ± 1.74	0.217
FPG (mmol/L)^a^	8.00 (6.10, 13.00)	9.00 (7.00, 13.60)	8.60 (6.00, 12.00)	0.534
FINS (mmol/L)^a^	10.25 (6.00, 16.00)	8.00 (5.00, 22.00)	12.00 (5.25, 18.00)	0.829
C-peptide (*μ*g/L)^a^	2.065 (1.35, 2.78)	1.54 (0.96, 2.24)	1.78 (1.13, 2.83)	0.349
HOMA2-IR^a^	5.78 (3.77, 8.06)	4.37 (3.07, 8.00)	4.72 (3.14, 7.91)	0.852
HOMA2-*β*^a^	120.90 (52.90, 192.40)	81.50 (45.02, 153.40)	101.70 (43.70, 199.25)	0.281
Comorbidity				
Hypertension, *n* (%)	22 (39.3%)	28 (41.8%)	13 (29.5%)	0.410
CHD, *n* (%)	5 (8.9%)	10 (14.9%)	7 (15.9%)	0.509
CVA, *n* (%)	3 (5.44%)	7 (10.44%)	8 (18.2%)	0.121

*Note:* Data are presented as means ± SD, median (interquartile range), or *n* (%).

Abbreviations: ALT, alanine aminotransferase; AST, aspartate aminotransferase; BMI, body mass index; CHD, coronary heart disease; CVA, cerebrovascular accident; eGFR, estimated glomerular filtration rate; FINS, fasting insulin; FPG, fasting plasma glucose; HbA1c, hemoglobin A1c; HOMA2-IR, homeostatic model assessment for insulin resistance; HOMA2-*β*, homeostatic model assessment for *β* cell function; NLR, neutrophil-to-lymphocyte ratio.

^a^Log transformed before analysis.

^#^The adjusted alpha by Bonferroni method is 0.0167.

^b^ *P* < 0.05 painless-DPN vs. non-DPN.

^c^ *P* < 0.05 painful-DPN vs. non-DPN.

**Table 2:** Logistic regression analysis of the association between spexin and DPN in all patients.

	**Serum spexin levels**			**P** **for trend**
	**Tertile 1**	**Tertile 2**	**Tertile 3**	
Median (pg/mL)	71.40	113.48	188.92	
Cases, *n*	56	57	54	
OR (95% CI) for				
Model 1	Ref.	0.31 (0.12–0.82)	0.11 (0.04–0.28)	< 0.001
Model 2	Ref.	0.29 (0.11–0.80)	0.11 (0.04–0.31)	< 0.001
Model 3	Ref.	0.31 (0.11–0.89)	0.10 (0.03–0.29)	< 0.001

*Note:* Data are presented as odds ratios (95% CI) for the risk of having overall DPN in all patients. Model 1: unadjusted. Model 2: adjusted for age, gender, and BMI. Model 3: model 2 further adjusted for duration of DM, C-peptide, and smoking status.

**Table 3:** Logistic regression analysis of the association between spexin and painful DPN in patients with DPN.

	**Serum spexin levels**			**P** **for trend**
	**Tertile 1**	**Tertile 2**	**Tertile 3**	
Median (pg/mL)	63.70	100.46	165.73	
Cases, *n*	37	37	37	
OR (95% CI) for				
Model 1	Ref.	0.20 (0.08–0.54)	0.08 (0.03–0.25)	< 0.001
Model 2	Ref.	0.22 (0.08–0.60)	0.07 (0.02–0.23)	< 0.001
Model 3	Ref.	0.22 (0.07–0.64)	0.06 (0.02–0.21)	< 0.001

*Note:* Data are presented as odds ratios (95% CI) for the risk of having pain in patients with DPN. Model 1: unadjusted. Model 2: adjusted for age, gender, and BMI. Model 3: model 2 further adjusted for duration of DM, C-peptide, and smoking status.

## Data Availability

The data sets in this study are available from the corresponding authors on reasonable request and wrote the manuscript.

## Abbreviations

DPN: Diabetic peripheral neuropathy
DRG: Dorsal root ganglion
BMI: Body mass index
sCr: Serum creatinine
HbA1c: Glycated hemoglobin
HOMA-IR: Homeostatic model assessment for insulin resistance
HOMA-*β*: Homeostatic model assessment for *β* cell function
i.p.: Intraperitoneally
STZ: Streptozotocin
NeuN: Neuronal nuclear protein
PBMCs: Peripheral blood mononuclear cells
TNF-*α*: Tumor necrosis factor-*α*
IL-6: Interleukin-6
MCP-1: Monocyte chemoattractant protein-1
FPG: Fasting plasma glucose
eGFR: Estimated glomerular filtration rate
NLR: Neutrophil-to-lymphocyte ratio
ALT: Alanine aminotransferase
AST: Aspartate aminotransferase
FINS: Fasting insulin
2hPBG: Blood glucose 2-h postprandial
HbA1c: Hemoglobin A1c
LPS: Lipopolysaccharide
CVA: Cerebrovascular accident
CHD: Coronary heart disease.

## Supporting Information

Supporting Information Additional supporting information can be found online in the Supporting Information section. Table S1 Correlation analysis between clinical characteristics and Spexin in all participants. Table S2 Baseline characteristics of participants in all patients without or with DPN according to tertiles of serum spexin level. The linear regression analyses were performed to estimate the association of spexin and variables. Table S3 Baseline characteristics of participants in DPN patients without or with pain according to tertiles of serum spexin level. The linear regression analyses were performed to estimate the association of spexin and variables.
