# Supplementary material for: Serum Spexin Level Is Negatively Associated With Peripheral Neuropathy and Sensory Pain in Type 2 Diabetes
Source: J Diabetes Res. 2024 May 23;2024:4538199. doi: 10.1155/2024/4538199 (PMC11199070; doi:10.1155/2024/4538199)
Supplement: Supporting Information — Additional supporting information can be found online in the Supporting Information section. Table S1 Correlation analysis between clinical characteristics and Spexin in all participants. Table S2 Baseline characteristics of participants in all patients without or with DPN according to tertiles of serum spexin level. The linear regression analyses were performed to estimate the association of spexin and variables. Table S3 Baseline characteristics of participants in DPN patients without or with pain according to tertiles of serum spexin level. The linear regression analyses were performed to estimate the association of spexin and variables. [file 4538199.f1.docx]

**Supplementary table 1.** Correlation analysis between clinical characteristics and Spexin in all participants

|  | Total | |
| --- | --- | --- |
|  | r | *P* |
| Age (years) | -0.131 | 0.091 |
| DM duration (years) | -0.100 | 0.199 |
| BMI (kg/m2) | 0.047 | 0.547 |
| NLR | -0.185 | 0.051 |
| ALT (U/L) | 0.146 | 0.060 |
| AST (U/L) | 0.2 | **0.010** |
| eGFR (mL/min/1.73 m^2^) | 0.027 | 0.731 |
| HbA1c (%) | -0.030 | 0.703 |
| FPG (mmol/L) | -0.106 | 0.175 |
| FINS (mmol/L) | 0.084 | 0.285 |
| C-peptide (μg/L) | 0.277 | **<0.001** |
| HOMA2-IR | 0.194 | 0.014 |
| HOMA2-β | 0.272 | **<0.001** |

**Supplementary table 2.** Baseline characteristics of participants in all patients without or with DPN according to tertiles of serum spexin level. The linear regression analyses were performed to estimate the association of spexin and variables.

|  | Serum spexin level | | | *P* for trend |
| --- | --- | --- | --- | --- |
|  | Tertile 1 | Tertile 2 | Tertile 3 |  |
| Median, pg/mL | 71.40 | 113.48 | 188.92 |  |
| Cases, n | 56 | 57 | 54 |  |
| Male, n (%) | 38(67.9%) | 42(73.7%) | 34(63.0%) | 0.589 |
| Age (years) | 63.70±12.21 | 61.58±8.60 | 58.31±12.60 | **0.013** |
| BMI (kg/m^2^) | 24.91±3.00 | 25.12±3.45 | 25.31±3.41 | 0.518 |
| Duration (years) ^a^ | 14.00(8.25, 20.00) | 10.00(5.50, 20.00) | 10.00(4.00, 20.00) | 0.152 |
| Smoking, n (%)^#^ | 22(39.3%) | 32(56.1%) | 20(35.1%) | 0.617 |
| NLR | 2.29(1.73, 3.12) | 2.35(1.40, 2.80) | 1.84(1.35, 2.55) | 0.313 |
| eGFR (mL/min/1.73 m^2^) | 97.33(78.69, 104.91) | 99.24(89.19, 106.91) | 96.48(83.54, 109.39) | 0.281 |
| Liver function |  |  |  |  |
| ALT (U/L) ^a^ | 17.00(13.00, 22.00) | 19.00(13.00, 30.00) | 18.00(14.00, 30.50) | **0.024** |
| AST (U/L) ^a^ | 16.00(13.00, 19.75) | 17.00(14.00, 20.05) | 18.50(14.75, 24.50) | **0.007** |
| Glucose metabolism |  |  |  |  |
| HbA1c (%) | 9.20±2.25 | 9.02±1.92 | 8.81±2.05 | 0.323 |
| FPG (mmol/L)^a^ | 9.10(6.90, 13.00) | 9.00(7.00, 14.00) | 7.95(6.00, 10.25) | 0.175 |
| FINS (mmol/L) ^a^ | 10.00(4.00, 18.00) | 9.00(6.00, 12.83) | 12.00(6.45, 21.25) | 0.202 |
| C-peptide (μg/L) ^a^ | 1.29(0.88, 1.97) | 1.94(1.39, 2.46) | 2.28(1.59, 3.13) | **0.002** |
| HOMA2-IR ^a^ | 3.47(2.48, 6.82) | 5.35(3.98, 8.01) | 6.14(3.90, 9.18) | **0.022** |
| HOMA2-β^a^ | 58.50(34.50, 131.85) | 109.05(55.50, 178.30) | 136.50(73.60, 203.05) | **0.002** |

**Supplementary table 3.** Baseline characteristics of participants in DPN patients without or with pain according to tertiles of serum spexin level. The linear regression analyses were performed to estimate the association of spexin and variables.

|  | Serum spexin level | | | *P* for trend |
| --- | --- | --- | --- | --- |
|  | Tertile 1 | Tertile 2 | Tertile 3 |  |
| Median, pg/mL | 63.70 | 100.46 | 165.73 |  |
| Cases, n | 37 | 37 | 37 |  |
| Male, n (%) | 26(70.3%) | 28(75.7%) | 27(73.0%) | 0.794 |
| Age (years) | 63.62±13.57 | 64.51±8.15 | 60.51±12.06 | 0.247 |
| BMI (kg/m^2^) | 24.46±2.90 | 25.08±3.81 | 24.95±3.19 | 0.529 |
| Duration (years) ^a^ | 13.00(7.00, 20.00) | 15.00(10.00, 20.00) | 17.00(7.50, 20.00) | 0.416 |
| Smoking, n (%)^#^ | 17(45.9%) | 15(40.5%) | 17(45.9%) | >0.999 |
| NLR | 2.30(1.75, 3.10) | 2.50(1.72, 3.36) | 2.01(1.58, 2.95) | 0.953 |
| eGFR (mL/min/1.73 m^2^) | 97.13(78.99, 105.51) | 99.30(77.58, 103.78) | 92.94(84.46, 109.67) | 0.332 |
| Liver function |  |  |  |  |
| ALT (U/L) ^a^ | 16.00(12.50, 23.00) | 20.00(13.00, 24.00) | 18.00(14.00, 26.50) | 0.219 |
| AST (U/L) ^a^ | 14.00(12.00, 19.00) | 17.00(15.00, 21.00) | 17.00(12.50, 24.00) | **0.037** |
| Glucose metabolism |  |  |  |  |
| HbA1c (%) | 9.15±2.23 | 8.86±1.96 | 9.09±2.08 | 0.905 |
| FPG (mmol/L)^a^ | 9.05(6.93, 13.98) | 9.80(6.50, 12.80) | 8.00(7.00, 11.00) | 0.436 |
| FINS (mmol/L) ^a^ | 12.00(5.00, 21.80) | 8.50(4.63, 16.88) | 9.00(6.00, 23.30) | 0.953 |
| C-peptide (μg/L) ^a^ | 1.31(0.83, 2.03) | 1.54(1.06, 2.76) | 1.82(1.27, 2.72) | 0.059 |
| HOMA2-IR ^a^ | 3.98(2.24, 7.81) | 5.47(3.02, 8.57) | 5.25(3.55, 7.72) | 0.201 |
| HOMA2-β^a^ | 61.40(39.00, 133.00) | 102.50(39.78, 192.23) | 121.95(60.93, 191.05) | **0.027** |
